# Competing demands on adult children: How do they shape their provision of informal care?

**DOI:** 10.1016/j.ssmph.2025.101754

**Published:** 2025-01-10

**Authors:** Edward Pomeroy, Francesca Fiori

**Affiliations:** a School of Geography and Sustainable Development, University of St Andrews, KY16 9AL, UK; b Department of Social Work & Social Policy, University of Strathclyde, G4 0LT, UK

**Keywords:** Ageing, Informal caring, Informal care model, MAIHDA

## Abstract

Population ageing is increasing the demand for informal care, heightening the importance of adult children as potential carers to their older parents. Adult children, however, may be subject to competing demands for informal care provision when individual characteristics, such as gender and employment status, combine with household level characteristics, such as the presence of young children or vulnerable family members. Previous research often considers these competing demands as separate factors which can influence the provision of informal care, rather than in combination. Therefore, this study exploits data from Wave 13 (2021–2023) of the UK Household Longitudinal Study and applies multicategorical multilevel analysis of individual heterogeneity and discriminatory accuracy (MAIHDA) to assess the additive and interactive role of competing demands in influencing the provision of informal care. The results indicate that the provision of informal care is driven by the additive influence of the competing demands. Moreover, they also reveal the layering of certain social characteristics, which cumulate, rather than intersect, to create a social profile with a notably higher predicted probability of providing informal care.

## Highlights

• We study informal care provision from adult children towards older parents.
•Intersecting individual and household factors create competing demands for care.
•We use multicategorical MAIHDA to quantify competing demands for informal care.
•Gender, employment and family roles all affect the provision of informal care.
•Their effect, however, is additive, rather than combined.

## 1 Introduction

Informal care can be defined as the provision of care and support to a family member, friend, or acquaintance with a chronic illness, disability, or other long-lasting care need due to ill health or ageing (Hoefman et al., 2013). Socio-demographic changes, including population ageing, will inevitably impact on the need for, and nature of, informal care (Agree & Glaser, 2009; Zarzycki et al., 2023). Whilst many older adults may experience healthy ageing, for others, advancing age is associated with heightened care needs due to an increasing prevalence of chronic health conditions, disabilities, and functional limitations (Abud et al., 2022; Hamblin & Lariviere, 2023). Kin relationships represent latent webs of support, indicating to the essential role family networks play in providing care to those in need (Arpino et al., 2021). This suggests that adult children form a salient part of the caring network towards their older adult parents who may have heightened care needs due to age-related vulnerabilities.

Adult children are a heterogeneous group, with the diverse combinations of their individual and household characteristics creating unique social positions. This diversity can shape caring experiences, as specific combinations of social characteristics may impact the likelihood of adult children providing care to their non-coresident parents. Despite the importance of examining the interaction between individual and household level characteristics, previous research has primarily focused on their independent effects in influencing the provision of informal care, rather than their combined impact (Hengelaar et al., 2023). Therefore, this study aims to explicitly investigate the role of overlapping combinations of social characteristics, at both the individual and household level, in the quantitative study of informal care provision.

## 2 Competing demands for informal care providers

This study is theoretically grounded in the Informal Care Model (ICM) (Broese van Groenou & Boer, 2016), whose three elements (needs, dispositional factors and context) can be used to analyse informal caring experiences. Whilst the ICM was initially developed to examine the onset of caregiving, the model can also be used to help explain the heterogeneity of informal care provision. Firstly, the ICM theorises that contextual factors, such as the social context surrounding the care provider, can enable or constrain the provision of care. Secondly, the model highlights that informal caring is triggered when someone is in need of care, often arising from an individual's physical or mental condition. Whilst care needs are not explicitly modelled in this study, they remain important to acknowledge as a key element of the ICM and a factor which is likely to trigger the provision of informal care.

The ICM then highlights that becoming a carer depends on dispositional factors, or the carers ability and willingness to take on caring tasks. The decision to provide care is multifactorial and is influenced by a range of dispositional factors, both at the individual and household level. This diversity creates unique social positions, highlighting the importance of examining these factors in combination, rather than in isolation. As such, this study draws inspiration from the recent advancements in critical quantitative research, developed primarily with the intent to quantify intersectional inequalities on various outcomes (Bauer, 2014; Evans, 2015; Holman & Walker, 2021). Whilst our analysis does not seek to interpret findings through the lens of systemic injustice or oppression, typical when engaging with intersectionality theory (Crenshaw, 1989, 1991), it draws from these methodological developments to investigate the potential for interactive effects of social characteristics. As such, we sometimes use the term "intersecting" to describe the combined influence of these characteristics in shaping the provision of informal care.

The consideration of time as a finite resource is the fundamental driver in restricting individuals' abilities to perform certain competing demands (Becker, 1965). Thus, the ability to provide informal care depends on the adult child's ‘true availability’, meaning that whilst they may be willing to provide care, they may be unable due to the competing demands they may face. As such, combining family and work responsibilities with informal care can be seen as two conflicting social roles with sometimes incompatible demands (Arksey, 2002; Plaisier et al., 2015). Although some individuals will be able to combine these social roles with informal care provision, others will not. Thus, a key combination of social characteristics to consider in relation to informal caring is how competing demands, both at the individual and household level, may impact on the likelihood of adult children providing care to their non-coresident parents.

Feminist scholars have widely recognised gender as an important factor associated with the provision of informal care, with a disproportionate involvement of women in informal caring (Calvó-Perxas et al., 2018; Batur et al., 2024). One potential explanation for the gendered nature of care provision is differences in the norms of obligation and responsibility towards family members (Finley et al., 1988; Silverstein et al., 2006). Research has shown that there are normative differences that typically exist between genders regarding the distribution of family demands and employment (McMunn et al., 2020; Vlachantoni et al., 2021). For example, there is often a distinct gender division in labour in which women are assigned the responsibilities of ‘homemaking’, such as caring for children, whilst men are responsible for ‘breadwinning’ through working (Hess et al., 2023; Sear, 2021).

Given changing demographic patterns, it is likely that an increasingly large group of adult children may become ‘sandwich caregivers’, simultaneously providing care to both their dependent children and older adult parents (Ansari-Thomas, 2024). Therefore, caring for dependent children is another competing demand within the household that adult children may face in addition to providing care to their parents. For example, research has shown that parenthood can constrain an adult child's ability to provide care (Szinovacz & Davey, 2013; Wiemers & Bianchi, 2015). Another factor to consider is an adult child's ability to share family related demands, such as caring for family members with care needs. Adult children who cohabit with a partner may be able to share some household tasks, in turn reducing the extent to which they experience multiple competing demands for care provision (Henz, 2006). As such, this implies that adult children who cohabit with a partner may face a less restrictive allocation of tasks among household members, potentially facilitating their ability to provide informal care to their parents (Byrne et al., 2006; Mentzakis et al., 2009).

Adult children's competing demands within the household, and the impact they may have on their ability to provide informal care, may also intersect with their employment status (Arber & Ginn, 1995). An adult child's ability to combine work and informal care could also depend on the characteristics of their job (Horrell et al., 2014; Plaisier et al., 2015). Previous research suggests that stopping work altogether or reducing work hours are the two most common strategies that informal carers adopt to continue their care provision (Gomez-Leon et al., 2019; Schmitz & Westphal, 2017). An adult child's employment status is a key competing demand to consider, as some individuals may be unable to reduce their labour supply, as this is often associated with reduced income and long-term negative consequences for career progression (Raiber et al., 2022; Urwin et al., 2022).

According to the relative resources theory, relative means, such as income, determine power dynamics within families, and in turn can influence the division of care work within them (Bracke et al., 2008; Civettini, 2015). Therefore, investigating how an adult child's income intersects with the other aforementioned competing demands for care provision is important. Multiple studies have found that caring obligations vary by socioeconomic status, with more socioeconomically disadvantaged groups having fewer opportunities to access formal care services (Brandt et al., 2022; Quashie et al., 2022; Schmitz et al., 2022). Furthermore, the existence of a formal care market enables substitution between informal and formal care, meaning that adult children with time costs lower than the cost of formal care, ceteris paribus, may choose to substitute informal caring for formal care provision (Pierre-Carl et al., 2010).

Overall, the extent to which adult children are likely to experience these competing demands for care provision is expected to differ among various dimensions of social characteristics, and whilst some may face multiple competing demands simultaneously, others will not. Therefore, it is essential to consider how factors such as gender, employment, other family caring responsibilities (including caring for dependent children and other individuals with care needs), the ability to share these competing demands with a partner and an individual's income, combine to produce differing experiences for adult children providing informal care to their non-coresident parents.

We expect each individual element of the competing demands to be important factors which may influence the likelihood of providing informal care. Additionally, we expect there to be interaction effects, as different combinations of competing demands are likely to exist across social groups which may differentially shape caring experiences. For example, the impact of an individual's employment status in shaping their caring experiences is likely to be influenced by their gender (Bauer & Sousa-Poza, 2015). Women, due to longstanding gender norms that position them as primary caregivers, are often expected to take on caring roles regardless of their employment status (Sarkisian & Gerstel, 2004; Haberkern et al., 2015). This is in contrast to men where societal norms typically place fewer expectations on them to adjust their work commitments for caring (Calasanti, 2010; Swinkels et al., 2017). Thus, these gendered dynamics highlight the potential interaction effect between gender and employment, with women more likely to be involved in caring than men, even when in the same employment status.

## 3 Data and methods

This study uses data from Wave 13 of the UK Household Longitudinal Study (University of Essex, Institute for Social and Economic Research, 2024), a nationally representative annual survey of the UK population. The question on the provision of informal care asks, ‘*Do you provide some regular service or help for any sick, disabled or elderly person not living with you?*’. Respondents are then asked a follow-up question regarding their relationship with the non-coresident person they provide care for, with a range of answer options including parent/parent-in-law. As the question does not distinguish between care provision towards parents or parents-in-law, in line with Zueras and Grundy (2024) we use the term parental caregiver to refer to those providing care to either of these relatives. Therefore, the outcome variable is a binary measure which indicates whether an adult child reported either ‘yes’ or ‘no’ to providing non-coresidential parental care. The sample was restricted to only include individuals who are aged 18–65, ensuring we capture those of working age and thus are those most likely to experience multiple competing demands for care provision. Secondly, as the focus of this study is on parental caregiving, the sample was further refined to only include adult children who had at least one parent alive outside the household and were therefore exposed to the possibility of providing care to them. Individuals with any missing values from the variables of interest were removed (n = 101), with checks showing that this did not change the distribution of the sample. This resulted in a total analytical sample of 11,835 working age adult children who have at least one non-coresident parent alive.

To conduct a quantitative appraisal of the intersecting role of competing demands for informal care provision, this study utilises multicategorical multilevel analysis of individual heterogeneity and discriminatory accuracy (MAIHDA). Developed as an extension of multilevel modelling, multicategorical MAIHDA enables researchers to go beyond traditional, single-dimension analyses by examining the simultaneous influence of multiple intersecting characteristics across a range of different outcomes (Evans, 2024; Jones et al., 2016; Rodriquez-Lopez et al., 2022). In MAIHDA, individuals are viewed as being nested within social strata, with strata treated as a type of context, analogous to how neighbourhoods or schools would be modelled in a standard multilevel framework (Anticona et al., 2022). MAIHDA involves two-level hierarchical regression models nesting individuals (level 1) within their social strata (level 2) (Evans, 2019). Thus, MAIHDA involves fitting two multilevel models, the null model which provides a detailed mapping of variations in the outcome between and within strata, and the main effects model which determines whether the effects are additive (i.e. the role of a single social characteristic) or multiplicative (i.e. the role of multiple intersecting social characteristics).

Evans et al. (2024) provide a comprehensive overview and tutorial of the MAIHDA approach, as well as its methodological advantages over traditional single-level regression approaches. As the outcome variable is a binary measure of care provision, logistic MAIHDA will be conducted, which estimates the probability of an adult child providing informal care within each social stratum. The full information on how the outcome and explanatory variables were (re)coded, and new variables created, can be seen in the supplementary materials (Table S1). All statistical analysis was conducted in R, and in line with Evans et al. (2024), maximum likelihood estimation was used to determine the best fitting parameters for the MAIHDA model.

Whilst MAIHDA approaches have been applied in a range of disciplines, such as epidemiology (e.g. Fisk et al., 2018; Holman et al., 2022) and educational research (e.g. Keller et al., 2023; Prior et al., 2022), to the best of our knowledge there has only been one study which has applied the method to the informal care context (Alonso-Perez et al., 2024). Using European longitudinal data, the authors investigate inequalities in the age of caregiving onset and the intersection between gender, migration background, education and occupation status. We add to their work by investigating how additional intersecting social characteristics at the individual and household level, here defined as competing demands, influence the provision of informal care. Therefore, this study leverages multicategorical MAIHDA to address the following research questions.

Research Question 1- Are competing demands a useful concept for predicting who is more likely to provide informal care?
Research Question 2- How do specific competing demands explain who is more likely to provide informal care?
Research Question 3- To what extent do intersecting competing demands influence the provision of informal care?
Research Question 4- Are differences in the provision of informal care more or less pronounced for certain groupings facing particular competing demands?

## 4 Results

Table 1 provides the percentage distribution of the variables used in the MAIHDA analysis. As evident, 10.7% of adult children reported providing non-coresidential parental care, whilst 89.3% of adult children remained uninvolved in the provision of care towards them. The table also provides the bivariate associations between the social strata defining variables and the dependent variable, with all six social strata defining variables having a statistically significant bivariate relationship with the dependent variable. This provides strong justification to their individual statistical relevance, alongside their theoretical relevance, to be included as a social characteristic used to generate the social strata. In this study, where six social characteristics are considered, each individual is assigned into a social strata which corresponds to a six digit ID code, where the digit positions were assigned in the following order (and categories): 1 = sex (1 = male, 2 = female), 2 = dependent children in the household (1 = no, 2 = yes), 3 = cares inside the household (1 = no, 2 = yes), 4 = cohabits (1 = no, 2 = yes), 5 = employment status (1 = employed full-time, 2 = employed part-time, 3 = self-employed or other) and 6 = income (1 = low, 2 = high). For example, this means that stratum one will be assigned ID code 111111, which represents a male, with no dependent children in the household, who does not provide care inside, does not cohabit, is employed full-time, and has a low income. Table 2 provides the sample size of the social strata, with the largest proportion of strata (30.7%) each having 100 or more individuals, whilst a notable proportion (26.1%) contain fewer than 10 individuals.

**Table 1:** Descriptive statistics.

Dependent Variable		N	%		

Care Outside					

	No	10,572	89.3%		
	Yes	1263	10.7%		

**Social Strata Variables**				Chi-squared	(P-Value)

Sex				69.7	<0.001
	Male	5009	42.3%		
	Female	6826	57.7%		
Dependent Children in Household				112.3	<0.001
	None	6368	53.8%		
	At least one	5476	46.2%		
Care Inside				33.6	<0.001
	No	11,219	94.8%		
	Yes	616	5.2%		
Cohabits				5.8	0.017
	No	2921	24.7%		
	Yes	8914	75.3%		
Employment Status				67.3	<0.001
	Employed full-time	5444	46.0%		
	Employed part-time	2783	23.5%		
	Self-employed or Other	3608	30.5%		
					
Income				17.6	<0.001
	Low	6959	58.8%		
	High	4876	41.2%		
					

**Control Variable**			Mean (SD)		

Age (years)			43 (11.6)	–	–

**Table 2:** Sample size of social strata.

Sample Size per Stratum	Number of Strata	% of Strata
100 or More	27	30.7
50 to 99	12	13.6
30 to 49	6	6.8
10 to 29	20	22.7
Less than 10	23	26.1
**Total**	**88**	

In addition, whilst age is not included as a social strata defining variable, we also control for the age of the adult child. Although this analysis does not directly model the care needs of recipients, controlling for the age of the care provider serves as an indirect control for the recipient's care needs. As data is not collected on the adult child's non-coresident parent/parent-in-law, this approach is under the assumption that older children are more likely to have older parents who in turn require higher levels of support (Pickard, 2013; Vlachantoni, 2017; Vlachantoni et al., 2011). In fact, our results show that for each year increase in the adult child's age the likelihood of providing informal care increases (OR = 1.10, p < 0.001), providing suggestive evidence in support of our assumption (Table 3).

**Table 3:** Parameter estimates for MAIHDA.

		Model A (Null Model)		Model B (Main Effects Model)	
		Odds Ratio [95% CI]	P-Value	Odds Ratio [95% CI]	P-Value
**Fixed-Effects: Regression Coefficients**					
Intercept		0.13 [0.11–0.15]	<0.001	0.00 [0.00–0.00]	<0.001
Sex	Male (Ref)				
	Female			1.77 [1.54–2.03]	<0.001
Dependent Children in Household	No (Ref)				
	At least one			0.86 [0.74–1.00]	0.044
Care Inside	No (Ref)				
	Yes			1.65 [1.31–2.08]	<0.001
Cohabits	No (Ref)				
	Yes			0.98 [0.84–1.15]	0.847
Employment Status	Employed full-time (Ref)				
	Employed part-time			1.22 [1.03–1.44]	0.020
	Self-employed or Other			1.00 [0.85–1.17]	0.961
Income	Low (Ref)				
	High			0.89 [0.77–1.03]	0.107
Age				1.10 [1.09–1.11]	<0.001
**Random Effects: Variances**					
Stratum-Level		0.32		0.00	
Individual Level		≈ 3.29		≈ 3.29	
**Summary Statistics**					
Variance Partition Coefficient (VPC)		8.92%		0.00%	
Proportional Change in Variance (PCV)				100%	
Number of Observations		11,835		11,835	

Notes: MLE estimation used for all models shown. 95% CIs shown in parentheses. VPC for logistic models are calculated using the latent response approach ($\sigma_{e}^{2}$ is set equal to the variance of the standard logistic distribution $\frac{\pi^{2}}{3}\approx 3.29$, where π denotes the mathematical constant 3.14159).

To examine the importance of the social strata for predicting who is more likely to provide informal care, we can determine the discriminatory accuracy of the social strata using the Variance Partition Coefficient (VPC) of the null model. Results show that 8.92% of the variance in the outcome variable is attributable to the strata level (Table 3). This value indicates that there are observable differences in the likelihood of providing of informal care across social strata.

The fixed-effect regression coefficients from the main effects model (Table 3) reflect the additive influence of the competing demands on the outcome. Therefore, these coefficients reflect how specific social characteristics explain who is more likely to provide informal care. The results indicate that women were more likely to have provided informal care than men (OR = 1.77, p < 0.001). Adult children with at least one dependent child inside their household were less likely to have provided informal care relative to those without dependent children (OR = 0.86, p < 0.001). Individuals who provided care inside their household, compared to those who did not, were more likely to have also provided parental care outside of their own household (OR = 1.65, p < 0.001). Adult children who are employed part-time, relative to those who were employed full-time were more likely to have provided informal care (OR = 1.22, p = 0.020). In contrast, no significant differences were observed between those in the self-employed or other employment status and those who were employed full-time in terms of the likelihood of providing informal care (OR = 1, p = 0.961). Results showed no discernible differences across adult children with different cohabitation statutes (OR = 0.98, p = 0.847) or incomes (OR = 0.89, p = 0.107), net of other social characteristics.

Whilst it is interesting to examine how individual social characteristics additively influence the provision of informal care, in this analysis we are also interested in examining how particular intersecting social characteristics may impact on the likelihood of adult children providing non-coresidential parental care. Therefore, to examine the extent to which intersecting competing demands influence the provision of informal care we can assess the adjusted VPC and the Proportional Change in Variance (PCV) values from the main effects model (Table 3). The VPC in the null model represents the upper bound of the explanatory power of the social strata as it includes the effect of the fixed-effects alongside the potential interactive effects of the variables that define the social strata (Evans, 2019). In contrast, the VPC in the main effects model represents the proportion of the total variance that remains (after adjustment for fixed-effects) that is attributable to interaction effects. The results reveal that the variance in the outcome attributable to the strata level reduced from 8.92% in the null model to 0.00% in the main effects model, resulting in a PCV value of 100%. This indicates that all the variance between strata is accounted for by the contributions of additive main effects.

**Fig. 1:**
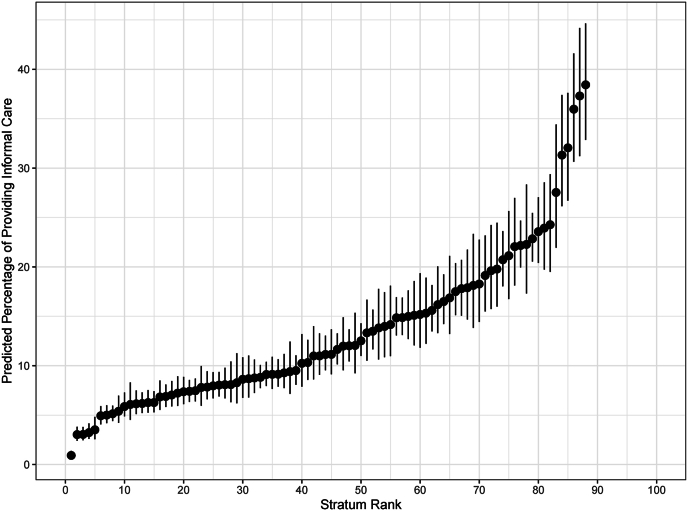
Predicted percentage of providing informal care. The graph shows the predicted percentage of individuals providing informal care across different strata, ranked by social stratum. The x-axis represents the "Stratum Rank”, while the y-axis displays the "Predicted Percentage of Providing Informal Care”.

**Fig. 2:**
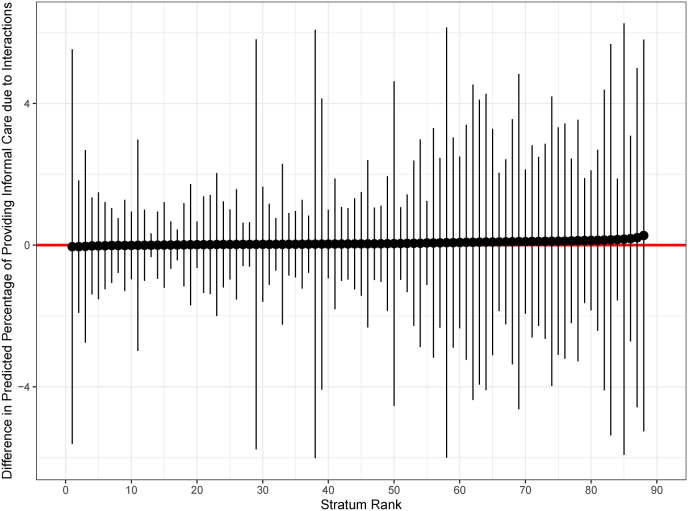
Difference in predicted percentage of providing informal care due to interaction effects only. Spikes indicate 95% CI. The graph shows the difference in the predicted percentage of providing informal care attributable to interactions across strata. The x-axis represents the "Stratum Rank”, while the y-axis represents the "Difference in Predicted Percentage of Providing Informal Care Due to Interactions”.

To determine if providing informal care is more or less pronounced for certain groupings facing particular competing demands, a list of the social strata with the highest and lowest predicted values can be generated by calculating the total predicted probability of providing informal care within each social stratum. The predicted values for the strata ranked from low to high is visualised in Fig. 1. The full table of predictions for each stratum to support this visualisation can be found in the supplementary materials (Table S2). Stratum 212221 (female, no dependent children in household, cares inside, cohabits, employed part-time, low income) has the highest predicted percentage of providing informal care (38.4%, CI [32.9%,44.6%]). This means that adult children in stratum 212221 have a 38.4% predicted probability of providing non-coresidential parental care. In contrast, stratum 112112 (male, no dependent children in household, cares inside, does not cohabit, employed full-time, high income) has the lowest predicted percentage of providing informal care (0.9%, CI [0.6%,1.4%]). In absolute terms this means individuals in stratum 212221 are 37.5 percentage points more likely to provide informal care compared to individuals in stratum 112112. The predicted probabilities attributable solely to interaction effects can be used to investigate whether differences in informal care provision are more or less pronounced in specific social strata (Fig. 2). However, in this context there are no significant interaction effects.

## 5 Discussion

### 5.1 Additive effects influencing informal care provision

The additive results from the MAIHDA reveal that specific social characteristics explain who is more likely to provide informal care. In line with findings from previous studies, the results show that women were more likely to provide informal care compared to men (Calvó-Perxas et al., 2018). Adult children with at least one dependent child inside their household were less likely to have provided informal care. This indicates that parenthood can constrain time to provide informal care, highlighting that the presence of dependent children is a key competing demand for informal care provision (Wiemers & Bianchi, 2015). Adults with dependent children are likely to be younger themselves and therefore balance multiple competing demands, including work and parenting, simultaneously (East, 2010). In contrast, older adult children are more likely to have fewer parenting and employment responsibilities potentially due to having older independent children and being less involved in time-consuming career building (Fast et al., 2020), in turn facilitating greater capacity to provide non-coresidential parental care. Additionally, it is important to consider not only the life course stage of these adult children, but also that of their parents, with the likelihood of providing non-coresidential parental care increasing as parents age.

In principle, adult children who care inside their household, and therefore face an additional competing demand for care provision, may be less likely to provide non-coresidential parental care. However, the results do not support this idea, with adult children who provided care inside their household actually being more likely to have provided parental care outside of their own household. It may be the case that adult children who already provide care inside their household generally exhibit a higher propensity to provide care, which can be explained by their norms and attitudes towards care provision (Bengston & Roberts, 1991). The experience of providing care within the household may reinforce and strengthen familial norms and attitudes towards care provision, making these individuals more likely to view care provision as an integral part of their role within their family (Burr et al., 2005; Vangen & Herlofson, 2023). An additional explanation for this finding could be that adult children who are already engaged in care provision within their household may have left employment or reduced their working hours, thereby gaining more flexibility in their schedules to provide additional care (Raiber et al., 2023).

In contrast to what may be expected, the results reveal that there were no significant differences in likelihood of providing care for those who cohabit with a partner or spouse, relative to those who do not. However, supplementary analysis focusing on the presence of siblings, another resource adult children may utilise to share care responsibilities in addition to their partner or spouse, reveals that adult children with at least one sibling alive are less likely to provide parental care (Table S3). Whilst not included in our theorisation as a competing demand, as the presence of siblings is a factor outside of the adult child's individual or household level, this result supports the idea that parental caring responsibilities can be shared within a larger family network and across siblings (Vergauwen & Mortelmans, 2019). Adult children who are employed part-time, relative to those employed full-time, were more likely to have provided parental care. This result illustrates the competing demands between employment and informal caregiving and aligns with the literature indicating that flexible working, such as reducing working hours, is often associated with involvement in care provision (Schmitz & Westphal, 2017).

In addition to flexibility in working hours, the rise of remote working during the COVID-19 pandemic has made working-from-home (WFH) arrangements a particularly timely and widespread form of flexible working that has been shown to facilitate care provision (Pomeroy & Fiori, 2024). As Wave 13 of the UKHLS introduced new variables which were not available in previous waves, such as the ability to measure WFH, it provided a unique opportunity to investigate the role of WFH in potentially facilitating care provision as society emerges from the pandemic context. An additional specification of the model, focusing on measuring WFH arrangements, reveals that no significant differences were observed between those who were employed and WFH, and those who were employed but did not WFH, in terms of the likelihood of providing informal care. Thus, this indicates that when emerging from the pandemic context, the benefits of WFH to informal care providers may not be fully observed, as the unique circumstances of the pandemic, rather than the ability to WFH per se, may have been the facilitator for care provision (Mutebi & Hobbs, 2022). Finally, the results indicate that there are no discernible differences across adult children with different incomes in terms of their likelihood to provide informal care. This suggests that income does not play a significant role, and factors such as familial or gender norms might override its influence in shaping the provision of informal care (Bengtson & Oyama, 2010; Hess et al., 2023).

### 5.2 Quantifying competing demands for informal care providers

The summary statistics from the main effects MAIHDA model reveal that the provision of informal care is fully accounted for by the additive effects, or in other words, the incremental effects of the individual social characteristics. The adjusted VPC value of 0.00% suggests that additive, rather than interaction effects, explain all the variation in the probability of providing informal care across different social strata. Furthermore, the lack of statistically significant interaction effects observed in this study indicates that each variable influences the provision of informal care independently, rather than in combination.

Despite finding no evidence of multiplicative effects, when analysing which social strata have the highest predicted percentages of providing informal care, a clear pattern emerges. The 7 strata with the highest predicted percentages of providing informal care (the most rightward points in Fig. 1) all share the same first three digits of their strata ID code, 212. This code corresponds to an adult child who is female, has no dependent children in the household and cares inside the household. This layering of additive effects indicates that this particular combination of characteristics creates a social profile in which adult children have a high predicted probability of providing non-coresidential parental care, highlighting the structural and normative dynamics that shape caring roles within families (Batur et al., 2024). The absence of dependent children can significantly reduce household responsibilities, freeing up time for other caring tasks, an effect that is especially pronounced for women who are most likely to assume child-caring duties (McMunn et al., 2020). Moreover, this aligns with broader societal expectations that position women as primary caregivers within families, including care provided inside the home (Vangen & Herlofson, 2023). Notably, this strata patterning persists irrespective of employment status or income, supporting the idea that women disproportionately take on caring responsibilities regardless of employment or financial status (Sarkisian & Gerstel, 2004; Haberkern et al., 2015).

## 6 Conclusion

Overall, this study aimed to quantify the importance of competing demands in influencing the provision of informal care. In doing so we theorised that multiple social characteristics, at both the individual and household level, may intersect to shape the likelihood of adult children providing care to their non-coresident parents. To empirically operationalise our theorisation of competing demands we leveraged multicategorical MAIHDA, to model both additive and interactive effects.

The results reveal that the provision of informal care is driven by the additive influence of the social characteristics, rather than by their combination. However, despite finding no evidence of multiplicative effects, the layering of additive effects highlights how certain social characteristics, in particular being a woman, without children and caring inside the household, creates a specific social profile in which adult children have a notably higher predicted probability of providing non-coresidential parental care. Whilst these results suggest that each social characteristic may independently increase the predicted probability of providing care, their cumulative impact can be substantial.

Despite the results suggesting that additive effects are more crucial than interactions between social characteristics in influencing the provision of informal care, the importance of considering social characteristics in combination, rather than in isolation, should not be dismissed. Whilst this study found no evidence of multiplicative effects, larger interaction effects could emerge when examining other aspects of care provision, such as the impact on caregivers' health or economic well-being. Additionally, larger combined effects could arise when considering different measurements of informal care. This study used a subjective measure of care provision, which whilst valuable for understanding personal perceptions towards care provision, may conceal certain inequalities by overlooking important elements such as the intensity or type of care provided. Therefore, investigating these more nuanced measures of care provision, and how they may differ based on intersecting social characteristics, is crucial for capturing the full range of caring experiences. Thus, more research is required to further investigate other potential interactive inequalities which arise in areas associated with informal care provision.

Furthermore, whilst this study has provided valuable insights into the provision of informal care, it does not investigate all possible intersecting social characteristics that may influence the provision of informal care. For example, whilst ethnicity could influence caring, its inclusion would expand the scope of this analysis beyond our current framework. Instead, this study focuses on the six key elements that we theorise to be related to competing demands for care provision, with a particular focus on both the individual and household level of the potential care provider. Our supplementary analysis focusing on the presence of siblings, a factor not included in our theorisation of competing demands as it is outside the potential care provider's household context, suggests that caring responsibilities can be shared across a larger network. As such, future research could extend our theorisation of competing demands to consider additional intersecting characteristics included within the broader network of the potential care provider-receiver dyadic unit (Burchardt et al., 2021).

By understanding how multiple social characteristics combine, we can in turn identify specific profiles of individuals who may be disproportionately involved in informal care provision. Recognising these groups is crucial, as those who may be unequally involved in informal caring may also be more likely to experience the consequences of providing care. Whilst not all impacts of providing care are negative (Pendergrass et al., 2019), many can adversely affect carers in a range of outcomes including on their health and economic well-being (Bauer & Sousa-Poza, 2015; Carers UK, 2023). These insights highlight a need for targeted policy interventions that provide support to the most impacted individuals, in order to prevent inequalities widening or newly developing for those who provide informal care (Oxfam Great Britain, 2024). Therefore, adopting an approach to research in which social characteristics are considered in combination provides more opportunity to analyse the complexity of informal care provision and in turn can contribute to the development of tailor-made support for informal carers, rather than generalised solutions (Hengelaar et al., 2023).

## CRediT authorship contribution statement

**Edward Pomeroy:** Writing – review & editing, Writing – original draft, Conceptualization, Formal analysis. **Francesca Fiori:** Writing – review & editing, Supervision, Conceptualization.

## Ethical statement

The study is based solely on the analysis of anonymised secondary data. It does not involve human participants or interaction with vulnerable population groups. Ethical approval was obtained from the University of St Andrews. Individual-level data from the UKHLS was downloaded from the UK Data Service website upon submission of an application form detailing the project objectives and intended data usage and the acceptance of the user agreement's terms and conditions. We abode by these strict rules, as defined in the user agreement. Outputs are presented in aggregate form and no information on individuals or individual households has been disclosed.

## Funding

This work was supported by the Economic and Social Research Council (ESRC) and Scottish Graduate School of Social Science (SGSSS) under Grant Number ES/P000681/1; and by the ESRC Centre for Population Change Connecting Generations research programme, under Grant Number ES/W002116/1.

## Declaration of competing interest

The authors declare that they have no known competing financial interests or personal relationships that could have appeared to influence the work reported in this paper.

## Data Availability

Data can be accessed through the UK Data Service (ukdataservice.ac.uk) upon signature of an End User Agreement. Statistical code will be made available upon request.
