# Supplementary material for: Competing demands on adult children: How do they shape their provision of informal care?
Source: SSM Popul Health. 2025 Jan 10;29:101754. doi: 10.1016/j.ssmph.2025.101754 (PMC11799968; doi:10.1016/j.ssmph.2025.101754)
Supplement: Multimedia component 1 [file mmc1.docx]

**Supplementary Material**

**Table S1:** How the outcome and explanatory variables were (re)coded, and new variables created for main MAIHDA model

| Variable Name | Survey Question(s) | Old | New |
| --- | --- | --- | --- |
| Age | N/A- Derived variable | Continuous variable | Used to restrict the sample to those aged 18-65 (i.e.. working age adults)  Control variable for MAIHDA |
| Living Relatives | 1. Excluding relatives who are living in this household with you at the moment, can you tell me which of these types of relatives you have alive at the moment? 2. Number of respondent’s biological/step/adoptive parents in household | 1. Mother, Father 2. None, One, Two | Used to restrict the sample to those who have at least one non-coresident parent alive |
| Care Outside | 1. Do you provide some regular service or help for any sick, disabled or elderly person not living with you? 2. Who is the first person that you look after or help? (What is their relationship to you?) 3. Who is the second person that you look after or help? (What is their relationship to you?) | 1. Yes, No 2. Parent/Parent-in-law, Grandparent, Aunt/Uncle, Other relative, Friend of Neighbour, Clients of voluntary organisation, Other 3. Parent/Parent-in-law, Grandparent, Aunt/Uncle, Other relative, Friend of Neighbour, Clients of voluntary organisation, Other | Yes:  If (1)=Yes and (2) and/or (3)= Parent/parent-in-law  No:  If otherwise |
| Sex | N/A- Derived variable | Male, Female | Male, Female |
| Dependent Children in Household | N/A- Derived variable  Number of own dependent children in the household. This variable uses the Department for Work and Pension (DWP)'s definition of a dependent child. | Continuous variable | Recoded into:   1. None 2. At least one |
| Care Inside | Is there anyone living with you who is sick, disabled or elderly whom you look after or give special help to (for example, a sick, disabled or elderly relative, husband, wife or friend etc)? | Yes, No | Yes, No |
| Cohabits | N/A- Derived variable  De facto Marital Status | Child under 16, Single and never married/in civil partnership, Married, In a registered same-sex civil partnership, Separated but legally married, Divorced, Widowed, Separated from civil partner, A former civil partner, A surviving civil partner, Living as couple | Yes:  If either: Married, In a registered same-sex civil partnership or Living as couple  No:  If otherwise |
| Employment Status | (1) Employee or self-employed  (2) Number of hours normally worked per week (employees only) | (1) Employee, Self-employed, Other  (2) Continuous variable | Recoded into:   1. Employed full-time for those working over 35 hours per week 2. Employed part-time for those working under 35 hours per week 3. Self-employed or Other |
| Individual Income | NA- derived variable.  Total net personal income is constructed as the sum of the six income components:  Component 1: Labour income+  Component 2: Miscellaneous income+  Component 3: private benefit income + Component 4: investment income +  Component 5: pension income +  Component 6: social benefit income | Continuous variable | Recoded into:   1. Low (defined when an individual’s income is below the mean value of the distribution, £1973) 2. High (defined when an individual’s income is equal or above the mean value of the distribution, £1973) |

**Table S2:** Predicted percentage by ranked strata of providing informal care

| **Rank** | **Stratum** | **Sex** | **Dependent children in HH** | **Care Inside** | **Cohabits** | **Employment Status** | **Income** | **n** | **Predicted %** | **Lower**  **95% CI** | **Upper 95% CI** |
| --- | --- | --- | --- | --- | --- | --- | --- | --- | --- | --- | --- |
| 1 | 112112 | Male | None | Yes | No | Employed FT | High | 1 | 0.9 | 0.6 | 1.4 |
| 2 | 121111 | Male | At least one | No | No | Employed FT | Low | 4 | 3 | 2.4 | 3.8 |
| 3 | 111121 | Male | None | No | No | Employed PT | Low | 85 | 3 | 2.5 | 3.8 |
| 4 | 121132 | Male | At least one | No | No | SE or Other | High | 4 | 3.2 | 2.6 | 4.1 |
| 5 | 112111 | Male | None | Yes | No | Employed FT | Low | 4 | 3.5 | 2.6 | 4.8 |
| 6 | 111111 | Male | None | No | No | Employed FT | Low | 238 | 4.9 | 4.1 | 5.9 |
| 7 | 121211 | Male | At least one | No | Yes | Employed FT | Low | 290 | 5 | 4.2 | 6 |
| 8 | 121212 | Male | At least one | No | Yes | Employed FT | High | 1130 | 5.1 | 4.4 | 5.9 |
| 9 | 112131 | Male | None | Yes | No | SE or Other | Low | 15 | 5.4 | 4.2 | 7 |
| 10 | 121112 | Male | At least one | No | No | Employed FT | High | 21 | 5.9 | 4.9 | 7.3 |
| 11 | 122132 | Male | At least one | Yes | No | SE or Other | High | 1 | 6.1 | 4.5 | 8.3 |
| 12 | 121232 | Male | At least one | No | Yes | SE or Other | High | 131 | 6.1 | 5.1 | 7.5 |
| 13 | 111112 | Male | None | No | No | Employed FT | High | 270 | 6.2 | 5.2 | 7.3 |
| 14 | 121222 | Male | At least one | No | Yes | Employed PT | High | 91 | 6.3 | 5.2 | 7.5 |
| 15 | 121231 | Male | At least one | No | Yes | SE or Other | Low | 328 | 6.3 | 5.3 | 7.4 |
| 16 | 221132 | Female | At least one | No | No | SE or Other | High | 33 | 6.8 | 5.5 | 8.5 |
| 17 | 111131 | Male | None | No | No | SE or Other | Low | 343 | 6.9 | 5.8 | 8.1 |
| 18 | 121221 | Male | At least one | No | Yes | Employed PT | Low | 85 | 7 | 5.9 | 8.5 |
| 19 | 121131 | Male | At least one | No | No | SE or Other | Low | 11 | 7.2 | 5.9 | 8.9 |
| 20 | 221131 | Female | At least one | No | No | SE or Other | Low | 119 | 7.4 | 6.1 | 8.9 |
| 21 | 221212 | Female | At least one | No | Yes | Employed FT | High | 478 | 7.4 | 6.4 | 8.5 |
| 22 | 221231 | Female | At least one | No | Yes | SE or Other | Low | 651 | 7.5 | 6.4 | 8.7 |
| 23 | 122211 | Male | At least one | Yes | Yes | Employed FT | Low | 17 | 7.8 | 6 | 10 |
| 24 | 221232 | Female | At least one | No | Yes | SE or Other | High | 82 | 7.8 | 6.6 | 9.4 |
| 25 | 221211 | Female | At least one | No | Yes | Employed FT | Low | 251 | 8 | 6.7 | 9.4 |
| 26 | 111211 | Male | None | No | Yes | Employed FT | Low | 341 | 8 | 6.9 | 9.4 |
| 27 | 111132 | Male | None | No | No | SE or Other | High | 44 | 8.1 | 6.7 | 9.8 |
| 28 | 122232 | Male | At least one | Yes | Yes | SE or Other | High | 4 | 8.1 | 6.3 | 10.4 |
| 29 | 112132 | Male | None | Yes | No | SE or Other | High | 1 | 8.3 | 6.2 | 11.2 |
| 30 | 122212 | Male | At least one | Yes | Yes | Employed FT | High | 45 | 8.6 | 6.8 | 10.8 |
| 31 | 122231 | Male | At least one | Yes | Yes | SE or Other | Low | 40 | 8.7 | 6.8 | 11 |
| 32 | 221112 | Female | At least one | No | No | Employed FT | High | 87 | 8.8 | 7.3 | 10.6 |
| 33 | 111212 | Male | None | No | Yes | Employed FT | High | 654 | 8.8 | 7.7 | 10.1 |
| 34 | 221221 | Female | At least one | No | Yes | Employed PT | Low | 809 | 9.1 | 7.9 | 10.4 |
| 35 | 221121 | Female | At least one | No | No | Employed PT | Low | 106 | 9.1 | 7.7 | 10.9 |
| 36 | 221222 | Female | At least one | No | Yes | Employed PT | High | 294 | 9.1 | 7.9 | 10.7 |
| 37 | 221122 | Female | At least one | No | No | Employed PT | High | 76 | 9.3 | 7.7 | 11.2 |
| 38 | 222132 | Female | At least one | Yes | No | SE or Other | High | 20 | 9.4 | 7.2 | 12.4 |
| 39 | 211111 | Female | None | No | No | Employed FT | Low | 340 | 9.5 | 8.1 | 11 |
| 40 | 122222 | Male | At least one | Yes | Yes | Employed PT | High | 4 | 10.2 | 7.9 | 13.2 |
| 41 | 111122 | Male | None | No | No | Employed PT | High | 29 | 10.3 | 8.6 | 12.6 |
| 42 | 222232 | Female | At least one | Yes | Yes | SE or Other | High | 18 | 11 | 8.6 | 14 |
| 43 | 221111 | Female | At least one | No | No | Employed FT | Low | 28 | 11 | 9.1 | 13.3 |
| 44 | 211112 | Female | None | No | No | Employed FT | High | 232 | 11.1 | 9.5 | 13 |
| 45 | 121121 | Male | At least one | No | No | Employed PT | Low | 2 | 11.1 | 9.1 | 13.7 |
| 46 | 211212 | Female | None | No | Yes | Employed FT | High | 494 | 11.6 | 10.1 | 13.3 |
| 47 | 222231 | Female | At least one | Yes | Yes | SE or Other | Low | 71 | 12 | 9.5 | 14.9 |
| 48 | 211211 | Female | None | No | Yes | Employed FT | Low | 410 | 12 | 10.4 | 13.7 |
| 49 | 122221 | Male | At least one | Yes | Yes | Employed PT | Low | 3 | 12 | 9.2 | 15.3 |
| 50 | 211131 | Female | None | No | No | SE or Other | Low | 369 | 12.5 | 11 | 14.3 |
| 51 | 222222 | Female | At least one | Yes | Yes | Employed PT | High | 10 | 13.3 | 10.5 | 16.7 |
| 52 | 111221 | Male | None | No | Yes | Employed PT | Low | 91 | 13.5 | 11.7 | 15.7 |
| 53 | 222122 | Female | At least one | Yes | No | Employed PT | High | 13 | 13.8 | 10.6 | 17.8 |
| 54 | 222211 | Female | At least one | Yes | Yes | Employed FT | Low | 16 | 14 | 10.9 | 17.4 |
| 55 | 222112 | Female | At least one | Yes | No | Employed FT | High | 7 | 14.2 | 11 | 18.1 |
| 56 | 111231 | Male | None | No | Yes | SE or Other | Low | 383 | 14.9 | 13.1 | 16.9 |
| 57 | 211121 | Female | None | No | No | Employed PT | Low | 246 | 14.9 | 13.1 | 16.9 |
| 58 | 211132 | Female | None | No | No | SE or Other | High | 48 | 15 | 12.7 | 17.6 |
| 59 | 222221 | Female | At least one | Yes | Yes | Employed PT | Low | 40 | 15.1 | 12.1 | 18.6 |
| 60 | 222131 | Female | At least one | Yes | No | SE or Other | Low | 19 | 15.2 | 11.8 | 19.4 |
| 61 | 222212 | Female | At least one | Yes | Yes | Employed FT | High | 19 | 15.3 | 12.2 | 18.9 |
| 62 | 111232 | Male | None | No | Yes | SE or Other | High | 136 | 15.6 | 13.5 | 18.2 |
| 63 | 121122 | Male | At least one | No | No | Employed PT | High | 3 | 16.2 | 13.3 | 20 |
| 64 | 211122 | Female | None | No | No | Employed PT | High | 57 | 16.5 | 14.2 | 19.2 |
| 65 | 212111 | Female | None | Yes | No | Employed FT | Low | 4 | 16.9 | 13.2 | 21.1 |
| 66 | 111222 | Male | None | No | Yes | Employed PT | High | 71 | 17.5 | 15.1 | 20.4 |
| 67 | 212121 | Female | None | Yes | No | Employed PT | Low | 7 | 17.8 | 15.1 | 20.7 |
| 68 | 212131 | Female | None | Yes | No | SE or Other | Low | 24 | 17.9 | 14.7 | 21.7 |
| 69 | 112121 | Male | None | Yes | No | Employed PT | Low | 1 | 18.1 | 13.8 | 23.3 |
| 70 | 222121 | Female | At least one | Yes | No | Employed PT | Low | 4 | 18.3 | 14.4 | 22.7 |
| 71 | 112212 | Male | None | Yes | Yes | Employed FT | High | 25 | 19.1 | 15.5 | 23.2 |
| 72 | 112221 | Male | None | Yes | Yes | Employed PT | Low | 4 | 19.6 | 15.7 | 24.2 |
| 73 | 112222 | Male | None | Yes | Yes | Employed PT | High | 5 | 19.8 | 16 | 24.5 |
| 74 | 211222 | Female | None | No | Yes | Employed PT | High | 129 | 20.7 | 18 | 23.6 |
| 75 | 112211 | Male | None | Yes | Yes | Employed FT | Low | 15 | 21.1 | 16.7 | 25.6 |
| 76 | 112232 | Male | None | Yes | Yes | SE or Other | High | 8 | 22 | 18.1 | 27 |
| 77 | 211231 | Female | None | No | Yes | SE or Other | Low | 536 | 22.2 | 19.9 | 24.7 |
| 78 | 122131 | Male | At least one | Yes | No | SE or Other | Low | 2 | 22.3 | 17.3 | 28.3 |
| 79 | 211221 | Female | None | No | Yes | Employed PT | Low | 486 | 22.8 | 20.5 | 25.4 |
| 80 | 211232 | Female | None | No | Yes | SE or Other | High | 73 | 23.6 | 20.4 | 27 |
| 81 | 112231 | Male | None | Yes | Yes | SE or Other | Low | 29 | 23.9 | 19.7 | 28.6 |
| 82 | 212211 | Female | None | Yes | Yes | Employed FT | Low | 13 | 24.3 | 19.5 | 29.4 |
| 83 | 212132 | Female | None | Yes | No | SE or Other | High | 3 | 27.5 | 21.9 | 34.4 |
| 84 | 212222 | Female | None | Yes | Yes | Employed PT | High | 7 | 31.3 | 26.1 | 37.4 |
| 85 | 212212 | Female | None | Yes | Yes | Employed FT | High | 10 | 32.1 | 26.7 | 37.6 |
| 86 | 212231 | Female | None | Yes | Yes | SE or Other | Low | 54 | 36 | 30.6 | 41.6 |
| 87 | 212232 | Female | None | Yes | Yes | SE or Other | High | 8 | 37.3 | 31.2 | 44.2 |
| 88 | 212221 | Female | None | Yes | Yes | Employed PT | Low | 25 | 38.4 | 32.9 | 44.6 |

**Table S3:** Parameter Estimates for sibship MAIHDA.

|  |  | **Model A (Null Model)** | | **Model B (Main Effects Model)** | | |
| --- | --- | --- | --- | --- | --- | --- |
|  |  | Odds Ratio  [95% CI] | P-Value | Odds Ratio  [95% CI] | | P-Value |
| **Fixed-Effects: Regression Coefficients** |  |  |  |  | |  |
| Intercept |  | 0.13  [0.11-0.15] | <0.001 | 0.00  [0.00-0.00] | | <0.001 |
| Sex | Male (Ref) |  |  |  | |  |
|  | Female |  |  | 1.77  [1.54-2.03] | | <0.001 |
| Dependent Children in Household | No (Ref) |  |  |  | |  |
|  | At least one |  |  | 0.86  [0.74-0.99] | | 0.042 |
| Care Inside | No (Ref) |  |  |  | |  |
|  | Yes |  |  | 1.65  [1.31-2.08] | | <0.001 |
| Cohabits | No (Ref) |  |  |  | |  |
|  | Yes |  |  | 0.98  [0.84-1.15] | | 0.812 |
| Employment Status | Employed full-time (Ref) |  |  |  | |  |
|  | Employed part-time |  |  | 1.22  [1.03-1.45] | | 0.019 |
|  | Self-employed or Other |  |  | 0.99  [0.84-1.17] | | 0.918 |
| Income | Low (ref) |  |  |  | |  |
|  | High |  |  | 0.89  [0.77-1.03] | | 0.108 |
| Age |  |  |  | 1.10  [1.09-1.11] | | <0.001 |
| Sibling Alive | No (Ref) |  |  |  | |  |
|  | At least one |  |  | 0.74  [0.62-0.89] | | 0.001 |
| **Random Effects: Variances** |  |  |  |  | |  |
| Stratum-Level |  | 0.32 |  | 0.00 | |  |
| Individual Level |  | $\approx$3.29 |  | $\approx$3.29 | |  |
| **Summary Statistics** |  |  |  |  | |  |
| Variance Partition Coefficient (VPC) |  | 8.92% |  | 0.00% | |  |
| Proportional Change in Variance (PCV) |  |  |  | 100% | |  |
| Number of Observations |  | 11,835 |  | 11,835 |  | |
